# Massive Early Post-Tracheostomy Hemorrhage From Inferior Thyroid Artery Branch Injury: An Oxygenation-Preserving Airway Rescue Strategy

**DOI:** 10.7759/cureus.116449

**Published:** 2026-09-17

**Authors:** Mahdi Abdallah, Ilker Ince, Mohamed Khalil, Alexis Cash, Ryan Staszak

**Affiliations:** 1 Anesthesiology, Penn State Health Milton S. Hershey Medical Center, Hershey, USA; 2 Anesthesiology and Perioperative Medicine, Penn State Health Milton S. Hershey Medical Center, Hershey, USA; 3 Surgical Critical Care/Trauma Surgery, Penn State Health Milton S. Hershey Medical Center, Hershey, USA

**Keywords:** difficult airway management, hemorrhage control, inferior thyroid artery injury, post-tracheostomy hemorrhage, surgical airway, video-assisted laryngoscopy

## Abstract

Early life-threatening hemorrhage following tracheostomy is uncommon and may arise from injury to branches of the inferior thyroid artery. Early recognition is critical because ongoing hemorrhage may rapidly flood and obscure the airway, compromise oxygenation, and make definitive airway management extremely challenging. We report a 70-year-old male with recent ischemic stroke who underwent tracheostomy and percutaneous endoscopic gastrostomy placement and developed massive bleeding from the tracheostomy stoma approximately 26 hours after the tracheostomy placement. During emergent airway management, temporary digital compression was applied to control hemorrhage while ventilation was maintained through the existing tracheostomy tube as oral endotracheal intubation was attempted using video laryngoscopy. After definitive endotracheal tube placement was confirmed, surgical exploration revealed hemorrhage from inferior thyroid artery branches requiring ligation and tracheostomy revision. This case highlights the importance of rapid recognition of early surgical-site arterial bleeding, preservation of oxygenation during airway rescue, and coordinated surgical hemorrhage control.

## Introduction

Tracheostomy is commonly performed in patients with severe neurologic injury requiring prolonged mechanical ventilation and enteral access following stroke or seizure [1]. Although minor bleeding may occur after tracheostomy, early life-threatening hemorrhage from arterial injury is rare and requires rapid recognition and coordinated airway management [2-4]. Delayed tracheo-innominate artery fistula (TIF) is the best-recognized cause of catastrophic post-tracheostomy hemorrhage; however, early arterial bleeding from injury to branches of the inferior thyroid artery represents a distinct and less frequently reported complication [5,6]. In contrast, early hemorrhage resulting from injury to branches of the inferior thyroid artery is rare and less frequently described [7,8]. We report a case of massive early post-tracheostomy hemorrhage secondary to inferior thyroid artery branch injury. This case emphasizes an airway management sequence that prioritizes preservation of oxygenation while definitive surgical hemorrhage control is achieved.

## Case presentation

A 70-year-old male was admitted after an ischemic stroke involving the right anterior and middle cerebral artery territories. At the time of the event, he was receiving aspirin 81 mg daily and prophylactic enoxaparin 40 mg daily. Concern for seizure activity at presentation required prolonged endotracheal intubation and enteral nutrition, prompting tracheostomy and gastrostomy planning prior to anticipated neurologic rehabilitation. The patient underwent surgical tracheostomy and percutaneous endoscopic gastrostomy placement with no immediate complications or evidence of bleeding.

Approximately 26 hours after surgical tracheostomy placement, the patient developed brisk, uncontrolled bleeding from the tracheostomy stoma and was emergently transferred to the operating room. Initial bedside measures, including direct pressure, tracheostomy cuff inflation, and digital compression through the tracheostomy stoma, were performed while the patient was prepared for emergent transfer to the operating room.

On arrival, active hemorrhage significantly limited visualization and raised concern for imminent airway compromise. Estimated intraoperative blood loss was 200 mL; however, the volume of blood loss prior to arrival in the operating room could not be quantified. Management focused on maintaining oxygenation through the existing tracheostomy tube while temporary hemorrhage control was achieved with digital compression, allowing definitive oral endotracheal intubation before surgical exploration. Continued digital compression through the tracheal stoma was achieved by placing a finger superior to the tracheostomy within the stoma, slowing the bleeding while preserving airway access and allowing a more controlled approach to definitive airway management. Given the severity of the hemorrhage, the neck and upper chest were prepared in anticipation of possible thoracic exploration if proximal vascular control became necessary; however, local exploration successfully identified and controlled the bleeding source.

Initial airway management involved oral endotracheal intubation with a 7.5-mm endotracheal tube using video laryngoscopy while maintaining ventilation through the existing 8.0 Shiley tracheostomy tube. After the endotracheal tube was believed to be correctly positioned by video laryngoscopy, the tracheostomy tube was removed; however, sustained end-tidal CO₂ was not detected, raising concern for failed or mispositioned intubation. The tracheostomy tube was promptly reinserted to re-establish ventilation while airway troubleshooting was performed. Further suctioning revealed significant blood pooling around the vocal cords. Hemorrhage was present both externally around the tracheostomy stoma and within the airway, impairing glottic visualization during intubation. After airway clearance, a second attempt of video-assisted endotracheal intubation was performed. This time, correct endotracheal tube placement was confirmed by sustained end-tidal CO₂ waveform, bilateral breath sounds, and fiberoptic bronchoscopy confirmation of tracheal placement, after which the tracheostomy tube was removed.

Surgical exploration demonstrated active bleeding from branches of the inferior thyroid artery adjacent to the tracheostomy stoma. Hemorrhage was controlled via suture ligation, followed by tracheostomy revision to an anatomically optimized and hemostatic position. No further bleeding was observed following irrigation and reassessment of the wound. Flexible bronchoscopy was then performed to remove blood clots from the right and left main bronchi before placement of a new 8.0 Shiley tracheostomy tube. Clot burden was greatest in the right upper lobe, and no active endotracheal bleeding was identified following clot removal. The patient received two units of packed red blood cells intraoperatively in the setting of acute hemorrhage, including unquantified blood loss prior to arrival in the operating room. Hemoglobin was 11.2 g/dL preoperatively and increased to 12.6 g/dL following transfusion at the conclusion of the procedure. Oxygen saturation remained between 95% and 100% throughout airway management. Exhaled tidal volumes increased from approximately 300 mL during the initial airway intervention to greater than 500 mL after definitive airway control was established, consistent with improved ventilation. Mean arterial pressure was initially in the low 60-mmHg range but improved to the 90-mmHg range following hemorrhage control. Initial sinus tachycardia resolved to normal sinus rhythm with heart rates ranging from 80 to 90 beats/min. Postoperatively, the patient remained hemodynamically stable. Multidisciplinary care, including physical and occupational therapy, was resumed, and neurologic secondary prevention planning was completed prior to discharge to a long-term acute care hospital (LTACH) with stable neurologic status.

## Discussion

The timing of hemorrhage after tracheostomy provides important diagnostic guidance. Bleeding occurring within the first 24 hours is most commonly related to surgical trauma, inadequate hemostasis, or injury to adjacent vascular structures [4]. In contrast, late hemorrhage occurring days to weeks after tracheostomy raises concern for major vascular erosion, such as TIF, which carries a high mortality despite intervention [6]. Early recognition of these surgical sources is critical because ongoing hemorrhage may rapidly compromise oxygenation, obscure the airway, and necessitate prompt surgical exploration for definitive hemostasis. Distinguishing early surgical bleeding from delayed fistulous complications is therefore essential, as the former is typically amenable to direct surgical exploration and ligation, whereas the latter often requires emergent temporizing maneuvers and vascular repair.

The inferior thyroid artery typically arises from the thyrocervical trunk and ascends medially toward the lower thyroid pole, giving off branches that supply the cervical trachea [7]. Because surgical tracheostomy is commonly performed at the level of the second and third tracheal rings, these branches may be vulnerable to injury during stoma creation or revision. In the present case, surgical exploration confirmed hemorrhage from ITA branches adjacent to the tracheostomy site, emphasizing the importance of recognizing this potential vascular source in early postoperative bleeding. Similar cases of post-tracheostomy hemorrhage arising from aberrant or injured inferior thyroid artery branches have been reported in the literature [8].

Massive post-tracheostomy hemorrhage creates competing priorities: preservation of oxygenation, maintenance of ventilation, and rapid hemorrhage control. Temporary control of bleeding was achieved with digital compression through the tracheal stoma, allowing airway access to be maintained while definitive airway management was pursued. Difficult airway guidelines emphasize oxygenation as the primary objective before definitive airway manipulation [9]. Maintaining the tracheostomy tube in situ during initial airway management allowed continued ventilation while definitive endotracheal intubation was attempted. After the first attempt failed to produce end-tidal CO₂, reinsertion of the tracheostomy tube restored ventilation while blood obscuring the glottis was suctioned and visualization improved. Subsequent video-assisted intubation allowed secure airway establishment and facilitated surgical hemorrhage control. The airway management sequence in this case consisted of temporary digital compression to reduce hemorrhage while maintaining ventilation through the existing tracheostomy tube, attempted oral endotracheal intubation while leaving the tracheostomy tube in place, immediate reinsertion of the tracheostomy tube when sustained end-tidal carbon dioxide was absent, repeat intubation after suctioning improved visualization, and removal of the tracheostomy tube only after definitive endotracheal tube placement had been confirmed. This sequence prioritized maintenance of ventilation while facilitating definitive surgical hemorrhage control.

Although video laryngoscopy was used for initial oral intubation in this case, active airway bleeding and blood pooling around the glottis impaired visualization and contributed to difficulty with airway placement. The blood surrounding the glottis became evident during subsequent airway suctioning and visualization; however, the reason it was not identified during the initial attempt cannot be determined retrospectively. Ongoing hemorrhage and accumulation of blood during airway manipulation may have contributed to the change in visualization between attempts. The rationale for not using flexible bronchoscopy during the initial intubation attempt was not documented. In a patient who remains adequately oxygenated and ventilated through an existing tracheostomy tube, bronchoscopic guidance may provide an alternative approach by allowing visualization and suctioning of blood and clots before the removal of the tracheostomy tube and the advancement of an endotracheal tube. In retrospect, this approach may have facilitated airway control during the initial attempt; however, significant active bleeding may also impair bronchoscopic visualization and suctioning. The approach used in this case should therefore be viewed as an adaptive response to an evolving airway emergency rather than a preferred or universally applicable airway management strategy. Video laryngoscopy was used in this case to facilitate glottic visualization during oral endotracheal intubation while ventilation was maintained through the existing tracheostomy tube. Although video laryngoscopy has demonstrated improved glottic visualization and intubation success compared with direct laryngoscopy in difficult airway scenarios [10], its effectiveness in this case was limited by active bleeding and blood pooling around the glottis. This case illustrates the importance of early recognition of surgical bleeding, preservation of oxygenation during airway rescue, and definitive surgical hemorrhage control in the management of early post-tracheostomy hemorrhage.

## Conclusions

Early hemorrhage following tracheostomy should prompt consideration of local arterial injury. This case illustrates the importance of maintaining oxygenation while securing definitive hemorrhage control and highlights the need to adapt airway management to the evolving clinical situation. Awareness of this uncommon presentation may facilitate timely diagnosis and appropriate multidisciplinary management.
